# CYP17 T27C polymorphism and prostate cancer risk: a meta-analysis based on 31 studies

**DOI:** 10.1016/S1674-8301(10)60033-4

**Published:** 2010-05

**Authors:** Bingbing Wei, Yunyun Zhang, Bo Xi, Junkai Chang, Jinming Bai, Jiantang Su

**Affiliations:** a Department of Urology, the First Affiliated Hospital of Nanjing Medical University, Nanjing 210029, Jiangsu Province, China; b Department of Neurology, the First Affiliated Hospital of Nanjing Medical University, Nanjing 210029, Jiangsu Province, China; c Graduate School, Peking Union Medical College, Beijing 100730, China; d Department of Epidemiology, Capital Institute of Pediatrics, Beijing 100020, China

**Keywords:** CYP17, prostate cancer, meta-analysis

## Abstract

**Objective:**

The cytochrome P450 17α-hydroxylase (CYP17) plays a vital role in androgen biosynthesis. A T-to-C polymorphism in the 5′ promoter region of CYP17 has been implicated as a risk factor for prostate cancer, but the results of individual studies are inconclusive or controversial. To derive a more precise estimation of the relationship, we performed an updated meta-analysis from 31 studies based on 27 publications.

**Methods:**

A comprehensive search was conducted to examine all the eligible studies of CYP17 polymorphism and prostate cancer risk. We used odds ratios (ORs) with 95% confidence intervals (CIs) to assess the strength of the association.

**Results:**

Overall, individuals with CC/CT genotype were not associated with prostate cancer risk (CC vs. TT: OR = 1.03, 95% CI = 0.86-1.24, *P* = 0.72, *P*_heterogeneity_ < 0.0001; CT vs. TT: OR = 0.99, 95% CI = 0.87-1.12, *P* = 0.88, *P*_heterogeneity_ = 0.0006). In the stratified analysis by ethnicity, there was a significantly increased risk of prostate cancer among individuals of African descent under the recessive model (OR = 1.56, 95% CI = 1.01-2.39, *P* = 0.04, *P*_heterogeneity_ = 0.65).

**Conclusion:**

This meta-analysis suggested that CYP17 polymorphism might be associated with prostate cancer risk among individuals of African descent.

## INTRODUCTION

Prostate cancer (PCa) is one of the most common causes of cancer-related death among men in Western countries[1]–[2]. Epidemiological data indicate that PCa is associated with advanced age, ethnicity, genetic and environmental factors[3]. Further, the development and progression of PCa are influenced by androgens[4]. Many studies have suggested that common germ line variation in genes related to androgens biosynthesis and metabolism could alter the function of these genes and the proteins they encode, thus altering PCa risk[5].

Genetic variation in one particular gene in this pathway, the cytochrome P450 17α-hydroxylase (CYP17), has been studied extensively in relation to gonadal development and the synthesis of androgens and estrogens[5]. CYP17 encodes an enzyme with both 17α-hydroxylase and 17, 20-lyase activities, the rate limiting steps in androgen biosynthesis. 17α-hydroxylase is responsible for hydroxylating pregnenolone and progesterone to their 17α-OH derivatives, which are then converted by 17, 20-lyase to dihydroepiandrosterone and androstenedione, and subsequently to testosterone and estrogens[6]. One single nucleotide polymorphism (SNP) rs743572 (denoted T(A1)27C(A2)), located 34 bp upstream from the translation start site of the gene[7], has been extensively studied in PCa[8]–[10]. However, results of studies that examined the association of the CYP17 T27C polymorphism to the incidence of PCa have been inconsistent[8]–[10]. Some studies have found that CYP17 polymorphism increases the risk of PCa, whereas others failed to confirm this observation.

Considering the possible small effect of the genetic polymorphism on PCa and the relatively small sample size in some studies, any small but real association may be underpowered, which would account for the apparent discrepancies among studies. In an attempt to resolve these contradictory results, a meta-analysis of all available studies[11]–[37] relating the T27C polymorphism of the CYP17 gene to the risk of developing PCa is presented here.

## MATERIALS AND METHODS

### Publication searched

PubMed was searched using the search terms: “CYP17”, “polymorphism” and “prostate cancer” or “prostate” (last search was updated on 5 May 2009). The search was limited to English-language papers. All studies matching the eligible criteria were retrieved, and their references of other relevant studies were hand-searched to find additional eligible studies. When more than one publication involved the same patient population, only the most recent or complete study was included in this meta-analysis.

### Inclusion criteria

The inclusion criteria were as follows: ① case-control studies; ② the distribution of CYP17 genotypes in prostate cancer cases and in a concurrent control group of the prostate cancer cases were eligible, regardless of whether they had a first-degree relative with prostate cancer or not; ③ disease-free controls, regardless of whether they had benign prostatic hyperplasia (BPH) or not; ④ sufficient published data for evaluating the OR with 95% CI.

### Data extraction

Two investigators independently extracted the data and reached consensus on all items. The following information was sought from each study: First author's surname, publication data, country, ethnicity of subjects, definition of cases, characteristics of controls, and the number of cases and controls with TT, TC and CC genotypes. Subjects were categorized as being of European, Asian and African descent. For studies in which subjects of different ethnicities were included, the data were analyzed separately according to the ethnicities for subgroup analysis. We did not define a minimum number of patients to include a study in this meta-analysis.

### Statistical methods

ORs with 95% CIs were used to assess the strength of association of CYP17 polymorphism with prostate cancer risk, according to the method of Woolf[38]. For CYP17 T27C polymorphism, we compared the risk of prostate cancer in the variant homozygote CC and in the heterozygote CT with the wild-type TT homozygote. The ORs with 95% CIs were calculated. The significance of the pooled OR was determined with a Z-test. In addition, genetic models assuming both dominant and recessive effects were used. Heterogeneity assumption was checked by the X^2^-based Q-test (a *P* value greater than 0.1 for Q-test indicates a lack of heterogeneity among studies)[39],[40], and the pooled OR estimate of the each study was calculated by the fixed-effects model. Otherwise, the random-effects model was used. The significance of the pooled OR was determined by the Z-test and *P* < 0.05 was considered as statistically significant.

In this meta-analysis, a subgroup analysis was performed to evaluate any ethnicity-specific effect. One-way sensitivity analysis was conducted to assess the stability of the results. Namely, a single study in this meta-analysis was deleted each time to reflect the influence of the individual data set to pooled OR.

An estimate of potential publication bias was carried out by funnel plot, using the standard error of log (OR). An asymmetric plot suggests a possible publication bias. Funnel plot asymmetry was assessed by the method of Egger's linear regression test, a linear regression approach to measure funnel plot asymmetry on the natural logarithm scale of the OR. The significance of the intercept was determined by the T-test suggested by Egger (*P* < 0.05 was considered representative of statistically significant publication bias).

All statistical tests for this meta-analysis were performed with STATA version 9.0 (Stata Corporation College Station, TX, USA), and the Review Manager Version 4.2 (The Cochrane Collaboration, Oxford, England).

## RESULTS

### Study characteristics

A total of 27 articles (including 31 studies) were retrieved based on the above search criteria for PCa susceptibility related to CYP17 T27C polymorphism. The main study characteristics are summarized in ***Table 1***. There were 6 studies of subjects of African descent, 11 studies of subjects of Asian descent, and 14 studies of subjects of European descent.

Prostate cancer was confirmed histologically or pathologically in 21 studies. Of the 31 studies, 9 studies used frequency-matched controls to the cases by age, or ethnicity. A classical polymerase chain reaction-restriction fragment length polymorphism (PCR-RFLP) assay was conducted in 25 studies, and a TaqMan assay was used in 4 studies. The genotype distributions among the controls of all but four studies were in agreement with Hardy-Weinberg equilibrium.

**Table 1:** Main characteristics of studies included in this meta-analysis.

First author (reference)	Year	Country	Ethnicity	Cases	Controls	Controls	
						*P*_HWE_^*^	Frequency of C allele
Wadelius(11)	1999	Sweden	European	178	160	0.137	0.438
Lunn(12)	1999	USA	European	96	159	0.811	0.343
Lunn(12)	1999	USA	African	12	8	0.719	0.313
Habuchi(13)	2000	Japan	Asian	252	131	0.545	0.512
Gsur(14)	2000	Austria	European	63	126	0.087	0.361
Chang(15)	2001	USA	European	225	182	0.435	0.360
Kittles(16)	2001	USA	African	71	111	0.932	0.297
Haiman(17)	2001	USA	European	590	782	0.118	0.386
Yamada(18)	2001	Japan	Asian	101	200	0.002	0.445
Latil(19)	2001	France	European	226	156	0.198	0.423
dos Santos-1(20)	2002	Brazil	European	84	128	0.221	0.344
dos Santos-2(20)	2002	Brazil	African	8	72	0.383	0.340
Stanford-1(21)	2002	USA	European	560	523	0.592	0.396
Stanford-2(21)	2002	USA	European	30	15	0.985	0.367
Tigli(22)	2003	Turkey	Asian	92	73	0.375	0.356
Lin CC(23)	2003	China	Asian	93	121	0.018	0.636
Madigan(24)	2003	China	Asian	174	274	0.222	0.608
Cicek-1(25)	2004	US	European	397	436	0.609	0.399
Cicek-2(25)	2004	US	African	38	38	0.165	0.329
Vesovic(26)	2005	Germany	European	174	89	0.095	0.393
Forrest(27)	2005	UK	European	262	462	0.348	0.359
Antognelli(28)	2005	Italy	European	384	360	0.058	0.444
Okugi(29)	2006	Japan	Asian	102	117	0.751	0.470
Yang(30)	2006	China	Asian	163	202	0.541	0.589
Sobti(31)	2006	India	Asian	100	100	0.634	0.300
Gunes(32)	2007	Turkey	Asian	148	102	0.713	0.284
Hamada(33)	2007	US	European	222	83	0.441	0.386
Cussenot(34)	2007	France	European	998	777	0.105	0.407
Onen(35)	2007	Turkey	Asian	100	105	0.910	0.433
Sobti(36)	2008	Indian	Asian	157	170	0.007	0.312
Sarma(37)	2008	US	African	126	322	0.000	0.387

*HWE: Hardy-Weinberg equilibrium

### Quantitative Synthesis

We observed a wide variation of the 27C allele frequencies across different ethnicities. The frequency of the 27C allele frequencies was 33.87% (95%CI=30.37-37.38) among controls of African descent, which was significantly lower than that in controls of European descent (38.85%; 95% CI=36.97-40.73, *P* = 0.013; ***Table 2***).

**Table 2:** Variant allele frequency of 27C in different ethnicities.

Ethnicity	No.comparisons (total sample size)	Controls
		Mean% (95% CI)
African^Δ^	6(851)	33.87(30.37-37.38)
Asian*	11(3077)	44.96(36.43-53.49)
European	14(8882)	38.85(36.97-40.73)

^*^Compared with European and African, *P* = 0.1481 and 0.0180, respectively. ^Δ^Compared with European, *P* = 0.0127

**Fig. 1:**
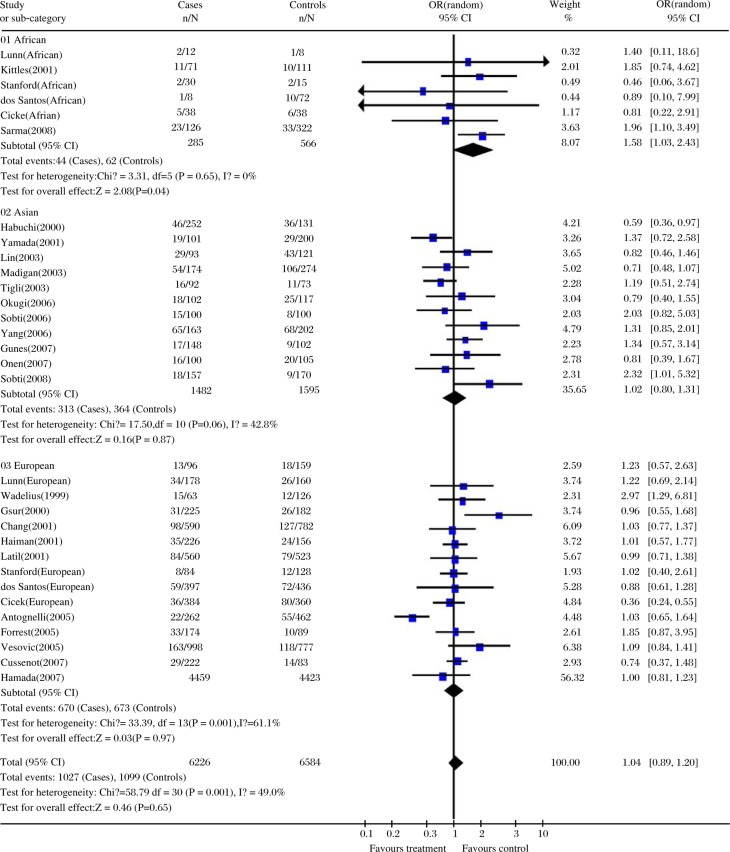
Forest plot of prostate cancer risk associated with the CYP17 T27C polymorphism (CC vs. CT+TT). The squares and horizontal lines correspond to the study-specific OR and 95% CI. The diamond represents the summary OR and 95% CI.

Overall, the CC and CT genotypes were not associated with significantly increased risk of prostate cancer risk (CC vs. TT: OR = 1.03, 95% CI=0.86-1.24, *P* = 0.72, *P*_heterogeneity_ < 0.0001; CT vs. TT: OR = 0.99, 95% CI=0.87-1.12, *P* = 0.88, *P*_heterogeneity_ = 0.0006; ***Table 3***). A similar negative association was maintained in dominant and recessive models (CC+CT vs. TT: OR = 1.00, 95% CI=0.88-1.14, *P* = 0.97, *P*_heterogeneity_ < 0.0001; CC vs. CT+TT: OR = 1.04, 95% CI=0.89-1.20, *P* = 0.65, *P*_heterogeneity_ = 0.001; ***Table 3***). In the stratified analysis by ethnicity, there was a significantly increased risk of prostate cancer among subjects of African descent in a recessive model (OR = 1.56, 95% CI=1.01-2.39, *P* = 0.04, *P*_heterogeneity_ = 0.65; ***Table 3***, Fig. 1).

**Table 3:** Stratified analysis of CYP17 polymorphism with prostate cancer risk.

Genetic model* (No.of studies) Overall(31)	Main effects of CYP17 polymorphism in cancer			
	OR(95% CI)	*P*	*P*_heterogeneity_	Analysis model^↓^
CC vs TT	1.03(0.86-1.24)	0.72	< 0.0001	R
CT vs TT	0.99(0.87-1.12)	0.88	0.0006	R
CC+CT vs TT	1.00(0.88-1.14)	0.97	< 0.0001	R
CC vs CT+TT	1.04(0.89-1.20)	0.65	0.001	R
Ethnic groups				
African(6)				
CC vs TT	1.51(0.95-2.40)	0.08	0.76	F
CT vs TT	1.27(0.66-2.42)	0.47	0.02	R
CC+CT vs TT	1.28(0.75-2.19)	0.37	0.06	R
CC vs CT+TT	1.56(1.01-2.39)	0.04	0.65	F
Asian(11)				
CC vs TT	1.09(0.74-1.62)	0.66	0.001	R
CT vs TT	1.07(0.81-1.42)	0.63	0.005	R
CC+CT vs TT	1.07(0.79-1.45)	0.66	0.0003	R
CC vs CT+TT	1.02(0.80-1.31)	0.87	0.06	R
European(14)				
CC vs TT	0.95(0.76-1.20)	0.68	0.002	R
CT vs TT	1.94(0.82-1.06)	0.31	0.07	R
CC+CT vs TT	0.98(0.82-1.07)	0.32	0.03	R
CC vs CT+TT	1.00(0.81-1.23)	0.97	0.001	R

*CC+CT *vs* TT: dominant model; CC *vs* CT+TT: recessive model. ^↓^F: fixed-effects model; R: random-effects model.

### Test of Heterogeneity

**Fig. 2:**
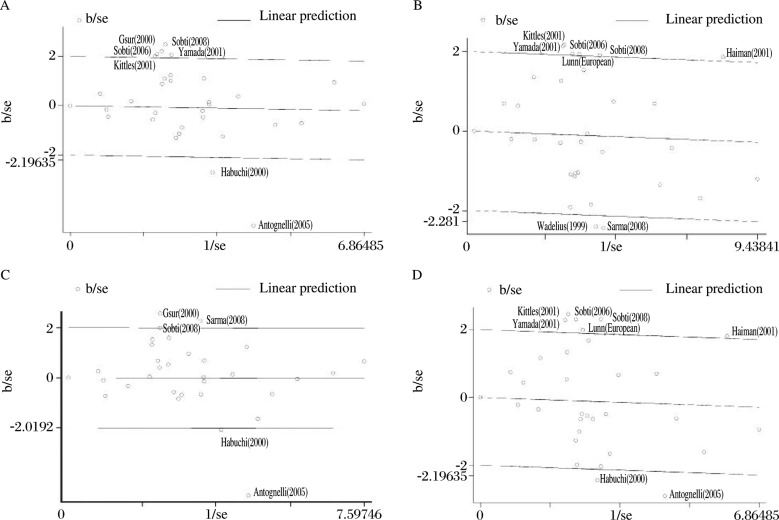
Galbraith plot analysis to evaluate heterogeneity. First author (year) identify the studies that lie outside the 95% CI. A: CC vs. TT; B: CT vs. TT; C: CC vs. CT+TT; D: CC+CT vs. TT

There was significant heterogeneity for homozygote comparison (CC vs. TT: *P*_heterogeneity_ < 0.0001), heterozygote comparison (CT vs.TT: *P*_heterogeneity_ = 0.0006), dominant model comparison (CC+CT vs.TT: *P*_heterogeneity_ < 0.0001), and recessive model comparison (CC vs.CT+TT: *P*_heterogeneity_ = 0.001). Although we assessed the source of heterogeneity by ethnicity, genotyping method, and sample size (more than 200 subjects in both cases and controls) for homozygote comparison, heterozygote comparison, dominant model comparison, and recessive model comparison, we did not find the source of heterogeneity by meta-regression. However, when Galbraith plots were used to evaluate the source of the heterogeneity, we found out the contributors of sources of heterogeneity for the various comparisons (***Fig. 2***).

### Sensitivity analysis

The influence of each study on the pooled ORs was examined by repeating the meta-analysis, omitting each study one at a time. This procedure did not significantly change the pooled ORs and influence the overall results (data not shown).

### Publication Bias

**Fig. 3:**
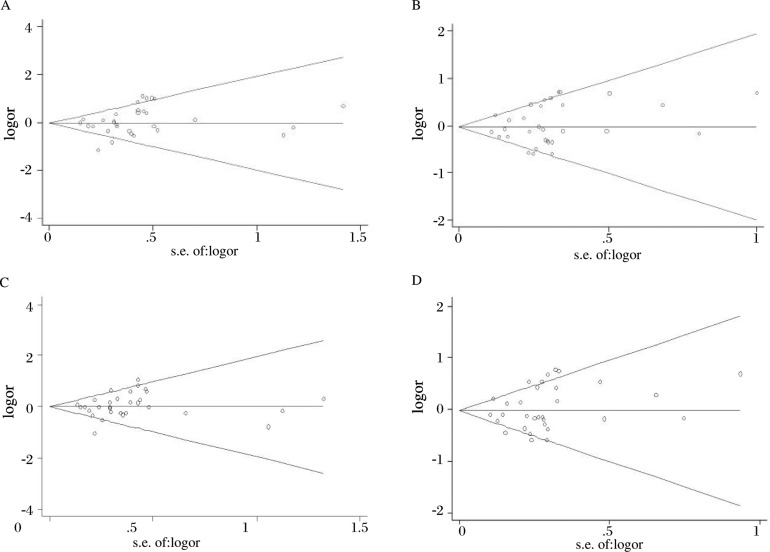
Funnel plot analysis to detect publication bias. Begg's funnel plot with pseudo 95% CI .A: CC vs TT; B: CT vs TT; C: CC vs CT+TT; D: CC+CT vs TT.

We used Begg's funnel plot and Egger's test to assess the publication bias of the literature used in this meta-analysis. The shape of funnel plots did not reveal any evidence of obvious asymmetry in all comparison models, and the Egger's test was used to provide statistical evidence of funnel plot symmetry. The results did not show any evidence of publication bias in ***Fig. 3*** (CC vs. TT: *P* = 0.164; CT vs. TT: *P* = 0.349; CT vs. CT+TT: *P* = 0.241; CC+CT vs. TT: *P* = 0.256).

## DISCUSSION

The present meta-analysis, including 6226 cases and 6584 controls from 31 published case-control studies, explored the association between CYP17 T27C polymorphism and prostate cancer risk. Several studies have shown that the CYP17 27C allele may be associated with increased risk of prostate cancer. Other studies, however, reported a decreased risk of prostate cancer in subjects with the CYP17 27C allele, or no association. Thus molecular epidemiological studies have presented seemingly contradictory results concerning a potential role of CYP17 T27C polymorphism in prostate cancer risk. In this study, we did not find that CYP17 T27C polymorphism was significantly associated with prostate cancer risk in a worldwide population. However, our analysis indicated that the C allele was significantly associated with prostate cancer among individuals of African descent under the recessive genetic model (OR = 1.56, 95% CI=1.01-2.39, *P* = 0.04, *P*_heterogeneity_ = 0.65). In addition, the variant allele frequency of 27C across the controls of African, Asian and European descent were 33.87% (95% CI: 30.37-37.38), 44.96% (95% CI=36.43-53.49) and 38.85% (95% CI=36.97-40.73) respectively. The result indicated that the distribution of 27C allele frequency showed a significant difference between individuals of African descent and individuals of Asian or European descent (*P* < 0.05), and the physiological function of 27C allele might differentiate among different ethnicities. It was suggested that there was a possible role of ethnic differences in genetic backgrounds and the environment they lived in. In Asians and Europeans, the influence of the 27C allele might be masked by the presence of other as-yet unidentified causal genes involved in prostate cancer development. Besides, it is also likely that the observed ethnic differences may be due to chance because studies with small sample size may have insufficient statistical power to detect a slight effect or may have generated a fluctuated risk estimate.

The results of this meta-analysis may be interpreted against the postulated biological context of CYP17 T27C polymorphism. It has been suggested that the CYP17 gene with three genotypes (TT, TC and CC) is known to mediate two key steps in sex steroid biosynthesis[41]. The C allele was considered to increase the transcription efficiency of the gene and improve enzymatic activity, which increased androgen synthesis, and finally resulted in an increased risk of PCa[14],[17],[42],[43]. However other studies found no differences in the levels of testosterone or other androgens and their metabolites (dehydrotestosterone, androstanediol glucuronide) in men based on their CYP17 genotypes[19],[44]. One study showed that the 27C allele created an additional Sp1-binding site in the CYP17 promoter region[7]. Subsequently, Sp1-binding at the T27C polymorphism site or within the promoter region of CYP17 in general could not be documented[42]. On the whole, no evidence is available to indicate that the 27C allele causes strong and consistent increased androgen levels. Given that androgens participate in the causal pathway of prostate cancer, the null effect of the CYP17 polymorphism on androgen levels would be consistent with the results of this meta-analysis.

Between-study heterogeneity may be attributed to many factors including the selection of publications, difference in population characteristics and sample sizes. We have addressed heterogeneity in these studies using a random-effects framework, which evaluates the variation in the pooled ORs based on individual OR of each study. Some limitations of this meta-analysis should be addressed. First, the association in this meta-analysis was investigated in all types of cases (hereditary, familial, or sporadic prostate cancer). There may be PCa-specific genetic effects among these cases but we could not obtain enough information to further estimate these effects. In addition, controls were not uniformly defined. Although most of the controls were mainly selected from healthy populations, some had BPH, and non-differential misclassification bias was possible because these studies may have included controls that had different risks for developing PCa. Second, lacking of the original information of the reviewed studies limited our further evaluation of potential interactions, because the interactions among gene-gene, gene-environment, and even different polymorphic loci of the same gene may modulate prostate cancer risk. Third, our result was based on unadjusted estimates, while a more precise analysis should be conducted if more detailed individual data were available, which would allow for an adjusted estimate by other factors, such as age. Lacking of information for data analysis may cause serious confounding bias. Fourth, misclassifications of disease status and genotyping may also influence the results because cases in several studies were not confirmed by pathology or other gold standard methods, and the quality control of genotyping was also not well-documented in some studies. In spite of these limitations, our meta-analysis also had some advantages. First, substantial numbers of cases and controls were pooled from different studies, which significantly increased the statistical power of the analysis. Second, the quality of case-control studies included in current meta-analysis was satisfactory and met our inclusion criteria. Third, we did not detect any publication bias, indicating that all pooled results should be unbiased.

In summary, this meta-analysis did not provide evidence of an association between CYP17 T27C polymorphism and prostate cancer risk in a pooled worldwide population, but under the recessive model this polymorphism was significantly associated with prostate cancer among individuals of African descent. However, additional large studies are warranted to validate our findings. Further, studies should use standardized unbiased genotyping methods and homogeneous cancer patients and well-matched controls. Moreover, more sophisticated gene-gene and gene-environment interactions should also be considered in further analyses, which should lead to a better, comprehensive understanding of the association between the CYP17 T27C polymorphism and prostate cancer risk.
